# Carbapenem Resistance: A Review

**DOI:** 10.3390/medsci6010001

**Published:** 2017-12-21

**Authors:** Francis S. Codjoe, Eric S. Donkor

**Affiliations:** 1 Department of Medical Laboratory Sciences (Microbiology Division), School of Biomedical & Allied Health Sciences, College of Health Sciences, University of Ghana, Korle Bu KB 143 Accra, Ghana; 2 Biomolecular Science Research Centre, Sheffield Hallam University, Sheffield S1 1WB, UK; 3 Department of Medical Microbiology, School of Biomedical & Allied Health Sciences, College of Health Sciences, University of Ghana, Korle Bu KB 143 Accra, Ghana; esampane-donkor@ug.edu.gh

**Keywords:** Gram negative bacilli, carbapenems, antibiotic resistance, carbapenemases

## Abstract

Carbapenem resistance is a major and an on-going public health problem globally. It occurs mainly among Gram-negative pathogens such as *Klebsiella pneumoniae*, *Pseudomonas aeruginosa* and *Acinetobacter* baumannii, and may be intrinsic or mediated by transferable carbapenemase-encoding genes. This type of resistance genes are already widespread in certain parts of the world, particularly Europe, Asia and South America, while the situation in other places such as sub-Saharan Africa is not well documented. In this paper, we provide an in-depth review of carbapenem resistance providing up-to-date information on the subject.

## 1. Introduction

Over seventy years the antimicrobial era has been marked by successive discoveries of a wide range of antibiotics and the subsequent emergence of antibiotic resistance. Bacterial resistance continues to increase, and drug researchers and manufacturing industries are not producing new drugs to replace the existing antimicrobials against which resistance has developed. The effects on current infection rates cannot be simply estimated [1,2]. The economic impact related to antimicrobial resistance was expected to cost over $105 billion annually worldwide and the largest relative economic impact was projected to be suffered by Africa with a drop in GDP of US $2895 billion in 2050, representing 20% of the region’s total economic output [3].

In recent times, development of antimicrobial resistance is rapidly changing, and the impending public health challenges these may cause in many health sectors need worldwide coordinated interventions. Many deaths have occurred as a result of this in Europe, and the European Centre for Disease Prevention and Control (ECDC) had estimated that 25,000 people may die each year from infections related to antimicrobial resistance [1,4]. Global reports documenting the infection burden of common and diverse bacterial pathogens that have developed resistance to available antimicrobial agents is alarming. Spellberg et al. [5] reported the annual costs of combating resistant bacterial infections to be between $21 billion and $34 billion in the USA alone.

Carbapenems possess broad spectrum antibacterial activity and have a unique structure that is defined by a carbapenem coupled to a β-lactam ring which confers protection against most β lactamases such as metallo-β-lactamase (MBL) as well as extended spectrum β-lactamases [6]. Consequently, carbapenems are considered one of the most reliable drugs for treating bacterial infections and the emergence and spread of resistance to these antibiotics constitute a major public health concern [7,8,9]. Recently, there has been a plethora of research information on carbapenem resistance, however, there are few comprehensive review papers discussing the research information. This paper therefore seeks to provide an in-depth review of carbapenem resistance providing up-to-date information on the subject.

## 2. Carbapenem Antimicrobials

Carbapenems are bactericidal β-lactam antimicrobials with proven efficacy in severe infections caused by extended spectrum β-lactamase (ESBL) producing bacteria [10]. There are a few examples, namely imipenem, meropenem, doripenem, ertapenem, panipenem and biapenem, in use worldwide as a result of the rising resistance to cephalosporin antimicrobials in the Enterobacteriaceae group. Recent emerging mechanisms of resistance accumulate through the spread of carbapenem-destroying β-lactamases leaving narrow therapeutic options [11]. The search for carbapenem agents was initially from diverse sources. Among these carbapenem agents, selection for treatment depends on the pathogen present.

### 2.1. Carbapenem Mode of Activity and Structure-Function Relationship

Carbapenems are potent members of the β-lactam family of antimicrobials that are structurally related to the penicillins. Mode of action of carbapenems is initiated first by penetrating the bacterial cell wall and binding to enzymes known as penicillin-binding proteins (PBPs) [12,13]. The main inhibiting series of PBPs are 1a, 1b, 2 and 3; and the resultant lethal effect is the inactivation of an inhibitor of autolytic enzymes within the cell wall which leads to the killing of the bacteria [14,15]. Earlier study reported that inhibition of PBPs 2 and 3 generally occurs in Gram-negative bacillus-shaped bacteria to form spherical cells and filamentous organisms, respectively [16].

Current understanding describes transpeptidase inhibition as the main enzyme target of carbapenems during bacterial cell wall synthesis. Through transpeptidation, a co-valent bond is formed by PBPs comprising of carboxypeptidase and transpeptidase enzymes which in effect prevent their peptide cross-linking activities during peptidoglycan biosynthesis. The lethal effects are thought to result in cell death by autolytic action within the bacterial cell [17]. According to [18], the mode of action of carbapenems remains uncertain. Much emphasis is placed on the rigid nature of the glycan backbone. Vitality of the cell wall is affected when PBPs are repressed, the glycan backbone weakens due to autolysis and eventually the cell is destroyed by osmotic pressure in Gram-negative bacteria [9,18].

Generally, carbapenems are preferred over other types of antimicrobials in treating invasive or life-threatening infections because of their concentration-independent killing effect on the infecting bacteria [19,20]. They are broad-spectrum and act against Gram-positive, Gram-negative bacteria and including anaerobes [13]. Notably, a wider spectrum of activity is found among the cyclic amine types of carbapenems with pyrrolidine derivatives namely meropenem, doripenem, panipenem and ertapenem. Exceptionally, biapenem also in the cyclic amine group, panipenem has a marginal effectiveness against Gram-negative bacterial strains. Imipenem/cilastatin and meropenem have been studied in comparative clinical trials establishing their efficacy in the treatment of a variety of infections including complicated intra-abdominal infections, skin and skin structure infections, community-acquired pneumonia, nosocomial pneumonia, complicated urinary tract infections, meningitis (meropenem only) and febrile neutropenia [13].

Comparatively, among the carbapenems, doripenem is highly stable against hydrolysis of most β-lactamases [21,22], but at the same time is described to have lower minimum inhibitory concentrations (MICs) with respect to meropenem and imipenem for *Acinetobacter baumannii* and *Pseudomonas aeruginosa* [23,24]. Evidence has shown that doripenem is less susceptible and much slower in carbapenemase hydrolysis (from 2 to 150 fold) than imipenem [25]. Ertapenem effectiveness against *Pseudomonas aeruginosa* is relatively limited when compared with meropenem or imipenem. Studies also confirmed that *Acinetobacter baumannii* has increased susceptibility to imipenem or doripenem compared with that of meropenem [18,26]. Several reviews on carbapenems and its carbapenemase-producing genes in Gram-negative bacteria have been reported.

### 2.2. Carbapenem Usage and Side Effects

Carbapenemases as a mechanism of resistance have been reviewed in the context of the effective use of carbapenems in combination therapy to enhance successes in patient outcomes [27]. Many enzymes are produced by bacteria that degrade β-lactam antimicrobials. Such enzymes are known as β-lactamases. However, bacteria find it difficult to effectively degrade carbapenem agents incorporated with β-lactamase inhibitors for in vivo use. The first compound to be used was imipenem, given with cilastatin, which inhibits the renal metabolism of imipenem and prolongs its half-life [28]. Imipenem, meropenem and doripenem have in vivo half-lives of approximately 1 h, while ertapenem has a half-life of approximately 4 h making it suitable for once-daily administration. Imipenem is noted for its dose-dependent gastrointestinal side effects as compared with the other carbapenems [13]. Ertapenem is the carbapenem that has the lowest activity against *Pseudomonas* species and other non-fermentative Gram-negative bacteria. Currently, different kinds of carbapenems are used in clinical practice as antipseudomonal agents; doripenem, imipenem and meropenem. The agents; ertapenem, imipenem and meropenem are poorly absorbed orally and require parenteral administration to be effective. Recently approved doripenem is of value among the current available carbapenems for treating serious infections [20,29].

Carbapenems have trivial hepatic metabolism effects leading to hepatotoxicity with jaundice, although this is an uncommon medical condition for these agents [13,30]. Most carbapenems are subject to degradation by the enzyme dehydropeptidase-1 (DHP-1) located in renal tubules and require co-administration with a DHP-1 inhibitor such as cilastatin. The later types of carbapenem agents including doripenem and ertapenem require no β-lactamase inhibitor as they are made stable in their mode of activity against Gram-negative bacterial infections. These compounds vary in their binding to PBP, thereby giving unique differences of activity towards different types of organisms. This can be seen in those that inhibit PBP3 and the opportunistic pathogen, *Pseudomonas aeruginosa*, as a specific example.

## 3. Development of Carbapenem Resistance

Infection with Carbapenem-Resistant Enterobacteriaceae (CRE) is emerging as an important challenge in health-care settings and a growing concern worldwide [31,32]. Carbapenem agents are very effective antimicrobials and administered intravenously with little or no allergic cross reactions in hospitals [33]. Each carbapenem agent has its own function, making its selection specific and delicate in clinical practice for serious infections [34]. The importance of the use of the carbapenems against Gram-negative pathogens cannot be overemphasized.

### 3.1. Intrinsic Resistance of Gram-Negative Bacilli

Development of resistance to carbapenems may be due to intrinsic or acquired resistance mechanisms or both. Large numbers of bacteria, both commensals and pathogens, naturally tend to be resistant to certain classes of antimicrobial agents. This insensitivity is termed intrinsic resistance, the occurrence of which limits and complicates drug selections for treatment. This can increase the risk for development of acquired resistance. For instance, Gram-negative organisms reduce the uptake of β-lactam drugs, by selectively altering their cell membrane porin channels. Reduction of outer membrane permeability in this manner prevents the β-lactams from reaching their targets.

Forsberg et al. [35] have shown transmission of different antimicrobial resistance genes from soil bacteria to clinical pathogens. Carriage of such genes was laterally exchanged to clinical pathogens and multiple mobilisation sequences including non-coding regions were identified in short-read sequence data from many soil bacteria [35]. For example, early MBLs studied and detected were chromosomal in nature from mainly opportunistic and environmental pathogenic bacteria including *Stenotrophomonas maltophilia*, *Bacillus cereus* and *Aeromonas* species [36]. These bacteria are only opportunistically pathogenic, and *Stenotrophomonas maltophilia* is the only organism commonly associated with frequent hospital acquired infections. Others in the same category, often considered commensals, typically carry chromosomal metallo-β-lactamase enzymes, which are not transferrable, from the groups which had a serine-based hydrolytic mechanism of action [37,38]. In the mid-1990s, chromosomal MBLs were detected in most carbapenem-resistant *Pseudomonas aeruginosa* and subsequently *Acinetobacter* species in clinical specimens. In recent times, MBL genetic materials are mobile in the Enterobacteriaceae family [11,39].

### 3.2. Acquired Resistance of Gram-Negative Bacilli

Bacteria have acquired multiple mechanisms of resistance including enzymatic inactivation, target site mutation and efflux pumps. Of these, the development and emergence of inactivating enzymes were established early following the discovery and clinical introduction of the β-lactam class of antibiotics. Over time, the β-lactam-hydrolysing enzymes extended their spectra of activity beginning with penicillinases, followed by cephalosporinases, then to ESBLs and most recently, to the MBLs and other carbapenemases. The MBLs have hugely impacted the utility of carbapenems (often considered as last resort drugs) which are used for the management of multi-resistant Gram-negative bacilli [40].

Many of the acquired carbapenemases found in Enterobacteriaceae are plasmid-mediated and there are several ways by which they can be spread amongst bacterial isolates. In addition, there are other important mechanisms conferring carbapenem-resistance that have been observed in recent times. First, the carbapenems that are resistant to hydrolysis can be destroyed in the presence of plasmid AmpCs in combination with ESBL enzymes, making Gram-negative bacteria insusceptible to carbapenem agents [41]. A significant number of ESBL genes have the potential to transfer between organisms. In contrast, those strains having their porins mutated or their expression modulated typically do not have potential for mobilisation but may proliferate locally within hospitals. This type of mechanism is recognisable in *Klebsiella*, *Enterobacter* species and *Escherichia coli* as well as other genera. Among the carbapenems, ertapenem is the worst affected; isolates may continue to be susceptible to other carbapenems. In most cases, reduced susceptibility to ertapenem depends largely on the degree of AmpC/ESBL presence and exact changes of the porins. The overexpression of efflux pumps and loss of *OprD* porin are the most common mechanism of carbapenem resistance in *Pseudomonas aeruginosa*, notably to imipenem. Other β-lactams may be affected by this mechanism. *Pseudomonas aeruginosa* efflux-pump overexpression occurs more regularly when meropenem is used when compared with imipenem. Both commensals and pathogenic bacteria have different mechanisms of using their efflux pumps to remove amphipathic or lipophilic substances in and out of the cells. These mechanisms have been recognised in other organisms such as *Enterobacter aerogenes* and *Klebsiella* species against imipenem agent [42].

Generally, Gram-negative bacteria are more resistant to a large number of antimicrobials and other chemotherapeutic agents than Gram-positive bacteria due to cell wall differences, external decreased membrane permeability, efflux pumps and the presence of various broad-spectrum-β-lactamases (e.g., ESBL and/or AmpC cephalosporinase). The resistance may be attributed to the presence of broad-specificity drug-efflux pumps [43,44]. The structural proteins involved in β-lactam resistance are sub-divided; including β-lactamases, PBPs and efflux pump systems.

One of the structures of drug-efflux pump systems extensively reviewed by Poole [45] was MexAB-OprM. This is a three-component pump; inner resistance-nodulation-division (RND) transporter ‘pump’ MexB, the outer membrane porin OprM and the soluble periplasmic MexA which acts strongly on a large number of antimicrobials, including β-lactams and its β-lactamase inhibitors (clavulanic acid as an example). Understanding of this three-way efflux pump and its structural features were reviewed by Eswaran et al. [46] and each component was investigated using *Escherichia coli* as an example; the orthologous inner membrane pump and outer membrane porin constituents AcrB and To1C have been resolved to 3.5 and 2.1 Å resolution respectively, while the structure of periplasmic component MexA from *Pseudomonas aeruginosa* has been solved to 3.5 Å resolution. The structural basis of the inner membrane pump, AcrB, has been determined in the presence of numerous hydrophobic small molecular compounds, which indicates a varied binding mode for each ligand, at least in this efflux pump element [47]. The three proteins; β-lactamase, PBP and efflux pump mechanisms interact to create a path to eliminate antimicrobial ligands. According to Kaatz [48] this has provided new ideas and understanding of target sites to drug development initiatives in future [48].

Thirdly, non-carbapenemase specific mechanisms such as mixture of porin loss in addition with specific efflux pump systems have been reported in *Acinetobacter* isolates. Weak detection levels have been found for Oxacillin-hydrolysing (OXA) carbapenemases, more than their absence [49,50]. Carbapenemase inhibition of carbapenems are of enormous clinical concern, however, resistance may be low level, compromising detection and reading of results for most hospital laboratories potentially having the consequence of increased patient mortality [51]. According to a report issued by Public Health England [50], carbapepems are known substrates for all carbapenemases, and undetected cases in the health care setting may cause complications when dealing with serious infections involving these enzymes [50].

### 3.3. Risk Factors for Acquisition of CRE Infection

There are a number of factors that predispose persons to infections by CRE and other multi-drug resistant (MDR) Gram-negatives including ESBL producers. Exposure to these resistant organisms can cause serious infections in patients with the following reported risk factors: immune-suppression, advanced age, admission to intensive care unit (ICU), mechanical ventilation, previous exposure to antimicrobials, organ or stem-cell transplantation and prolonged hospital stay [52,53]. With respect to antimicrobial agents, the risk of CRE acquisition appears to be much higher in the developing world particularly in sub-Saharan Africa where there is a predominance of irrational use of such drugs [54,55]. Health-care associated infections caused by CRE, mainly *Klebsiella pneumoniae,* have been encountered most commonly in ventilator-associated pneumonia, bacteremia, urinary tract and surgical site infections. Generally, *Klebsiella pneumoniae* carbapenemase (KPC)-producing carbapenemases in the β-lactamase molecular class A are rare, however, the variants are rapidly spreading, with KPC-2 and KPC-3 frequently reported in the UK [56]. The gene is carried on a transposon which increases the risk of spreading in many hospital settings extensively worldwide [57,58]. In clinical situations, where KPC-producing bacteria are a major concern, early intervention has to be taken to prevent death by administering effective empiric antimicrobials when the patient is immunocompromised, undergoing organ transplants or during cancer treatment [59,60].

There are reports which suggest that long-term care facilities play a crucial role in the spread of CRE. Neuner et al. [61] reported readmission of 72% patients who were discharged with carbapenem-resistant *Klebsiella pneumoniae* bloodstream infections at a hospital in Ohio, USA while Ben-David et al. [62] reported 42% in their initial hospital stay with records from post-acute care facilities in Israel. A recent systematic review on community-associated or community-onset CRE reported prevalence of 0–29.5% [63]. Growing evidence suggests early detection of CRE-colonized patients on admission to long-term care facilities may help to prevent institutional outbreaks and limit regional spread of this emerging public health threat. Despite the risk of CRE in long-term care facilities, some studies have reported very low prevalence of CRE at such facilitates and this could be due to several factors such as active surveillance and poor detection methods [64]. A prospective study in Central Virginia by Lewis et al. [65] concluded that CRE is more prevalent in regional acute care institutions than Long-term acute care hospitals (LTACHs) [65].

## 4. Molecular Classification of Carbapenemase Enzymes

A large variety of carbapenemases have been identified in *Enterobacteriaceae* belonging to 3 classes of β-lactamases: the Ambler classes A, B and D β-lactamases. These classes are of greatest clinical importance among nosocomial pathogens. Class A, C and D β-lactamases all share a serine residue in the active site. The clinical role of a fourth class (Ambler class C) is unknown but possesses some extended activity towards carbapenems [37,66].

### 4.1. Class A Carbapenemases

A number of this class of enzymes have been identified; some are chromosomally encoded—NmcA (not metalloenzyme carbapenemase A), SME (*Serratia marcescens* enzyme), IMI-1 (Imipenem-hydrolysing β-lactamase), SFC-1 (*Serratia fonticola* carbapenemase-1), with the others being plasmid encoded—KPC (KPC-2 to KPC-13), IMI (IMI-1 to IMI-3), derivatives (GES-1 to GES-20) of GES (Guiana extended spectrum), but all actively hydrolyse carbapenems and are partially inhibited by clavulanic acid [32,41]. Of these, the KPCs are the most prevalent and after a few years of its discovery, had spread worldwide and caused outbreaks in many Asian, North American and European countries as well as in Africa. KPC producers have evolved to be multidrug resistant to β-lactams thereby limiting therapeutic options for treating KPC-related infections in patients [67]. A review of KPC genes by Perez and van Duin [68] stated that there are currently 12 additional variants of *bla*KPC existing globally [68]. A study has shown that due to limited therapeutic options, KPC resistant clones are disseminating internationally in several areas that have different multi-locus locations with additional β-lactamase content which differ by size, number and structure of plasmids to compare producer isolates from different countries [56]. However, a single genetic element (transposon Tn4401) has been found in *bla*KPC genes [32,69]. For instance, between 2009 and 2010, Canadian Nosocomial Infection Surveillance Program investigated 7 cases of KPC-3-positives involving *Serratia marcescens* (1)*, Escherichia coli* (1), *Klebsiella oxytoca* (1) and *Klebsiella pneumoniae* (4) from a hospital and used multilocus sequence typing (MLST) to identify these organisms. This study revealed ST512 as a single-locus variant from the original known ST258 *Klebsiella pneumoniae* isolates widespread in Greece. The indication was that there was interspecies and intra-species were plasmid-mediated transfer belonging to the repFIIA replicon type. In another incidence, an elderly patient who visited Greece in 2012 contracted community acquired KPC-2-positive *Klebsiella pneumoniae*. This was isolated from the patient’s urine confirming it is widespread in Greece [70,71,72,73].

### 4.2. Class B Carbapenemases

These enzymes are mainly in the class of β-lactamases having the ability to hydrolyse carbapenems but are susceptible to inhibition by ethylenediaminetetraacetic acid (EDTA), a chelator of Zn^2+^ and other divalent cations. The mechanism of hydrolysis depends on interaction of the β-lactam drugs with zinc ions in the active site of the enzyme. The most common metallo-β-lactamase families include the New Delhi metallo-β-lactamase 1 (NDM-1), Imipenem-resistant Pseudomonas (IMP)-type carbapenemases, VIM (Verona integron-encoded metallo-β-lactamase), GIM (German imipenemase) and SIM (Seoul imipenemase). The genes encoding these enzymes are often located within a variety of integron structures and incorporated in to the gene cassettes [37,74].

By mid-2010, the NDM-1 gene may have been acquired by bacteria from the community and introduced to other countries including Europe and the United States through tourists travelling around the globe, while the same strains have been found in environmental samples in India [75,76]. At least, 8 variants have been described and identified in this group. NDM-genes are dominant in *Klebsiella pneumoniae* and *Escherichia coli* isolates but have also been found in association with *Acinetobacter baumannii* and *Pseudomonas aeruginosa* organisms [41,77,78].

Currently, up to 18 varieties of IMP-type carbapenemases have been identified. This type was first recognized in the 1990s in Japan. The majority of these enzymes were investigated in *Acinetobacter* and *Pseudomonas* species as well as those in the Enterobacteriaceae family. The IMP genes have also been found in Brazil and Canada after first reports from Europe in 1997. These gene types have spread slowly to other countries in the Far East and subsequently to the United States and Australia [37,79].

VIM genes are rarely encountered in Enterobacteriaceae but occur most commonly in *Pseudomonas aeruginosa* and *Pseudomonas putida*. Another growing family of carbapenemases first isolated in Verona, Italy, in 1997, and exhibited predominantly integron-associated metallo-β-lactamases. The family consists of 14 members. The VIM and IMP families have some similarities in terms of which plasmids they are carried on and that they are integron-associated. The VIM family has amino acid sequence diversity of up to 10%, 15% for IMP and 70% between the two families. Both hydrolyse all β-lactams except monobactams, and are susceptible to all β-lactam inhibitors [80].

### 4.3. Class D Carbapenemases

These enzymes are serine-β-lactamases poorly inhibited by EDTA or clavulanic acid. These carbapenemases are of the OXA enzyme type, and have a weak activity against carbapenems. The enzymes are found primarily in non-fermenter organisms such, *Acinetobacter baumannii*, *Pseudomonas aeruginosa* and rarely in isolates of Enterobacteriaceae family in most countries including the United Kingdom and in the United States [81,82]. The OXA β-lactamase with carbapenemase activity was first described by Paton et al. [83], and the enzyme purified from a multidrug-resistant *Acinetobacter baumannii* isolated in 1985 from a patient in Edinburgh, Scotland. Activity of OXA carbapenemases is increased by upstream elements which control gene expression [84]. The major concern with OXA carbapenemases is their ability to rapidly mutate and expand their spectrum of activity. Studies by Mathers et al. [85] reported frequent detection of class D among the Enterobacteriaceae family making this a threat and a major public health problem worldwide [85]. Currently, OXA-48 is the most common and is widespread in *Klebsiella pneumoniae* in Turkey, the Middle-East, North Africa and Europe [86]. Non-nosocomial OXA-24 type has been found in environmental *Acinetobacter* species while the spread of OXA-23 type which occurs globally, is more encountered in USA and Europe. The OXA-58 group had been described significantly over the globe in several outbreaks [87]. OXA-48–type producers are among the most difficult carbapenemase producers to be identified due to their point mutant analogues with ESBLs; thus, their true prevalence rates are difficult to estimate. Over the years, there have been 102 unique OXA sequences identified of which 9 are extended spectrum β-lactamases and at least 37 are considered to be carbapenemases [32,37].

## 5. Laboratory Detection of Carbapenem-Resistant Organisms

The presence of a carbapenemase can be detected by a number of methods in clinical laboratories. These include automated systems or disc diffusion, MICs, selective agar, modified Hodge test, synergy tests (e.g., E-tests or double disc tests), spectrometrics, whole genome sequencing and molecular methods. The majority of the genes controlling carbapenemase production are transferrable by plasmids. The presence of carbapenemases signifies some relevance to clinicians; however, care has to be taken in the management of patients to carbapenem therapy which may change depending on the mechanism of resistance existing at that time. Currently, detection of the enzymes is difficult because of the different mechanisms involved and unreliable techniques practised in some clinical laboratories [68]. On the other hand, extended-spectrum β-lactamase-inhibitors, such as clavulanic acid, tazobactam and sulbactam when used in combination with a carbapenem agent are also unreliable for phenotypically detecting carbapenemase production in bacterial isolates.

### 5.1. Phenotype-Based Methods

The baseline test that first predicts carbapenemase enzyme production is disc diffusion or the use of automated systems. Prior to this susceptibility testing, a bacterial species identification is required which takes about two days for common bacterial pathogens, and although it is highly accurate, there are inherent problems in differentiation between acquired and intrinsic resistance [88,89].

For disc diffusion, impregnated discs containing a standard amount of an antibiotic agent are placed on agar plate seeded with a bacterium to be tested. As the organism grows during overnight incubation, the antibiotic agent diffuses into the agar medium. Susceptibility of the test organism is proportional to the zone of inhibition produced by the antibiotic used. With the automated systems, instruments are used to analyse antibiotic susceptibility testing with standardised inoculum for the test strain and diluted in a specialised broth with a drop of antimicrobial susceptibility testing indicator added. The final inoculum turbidity is adjusted to 0.5 McFarland Standard. The inoculum is poured into a panel, closed in a secured place and the inoculated panel is then placed into, for example, instrument for VITEK or VITEK 2 automated system. The panel is then read automatically by the instrument, the data generated is analysed with preliminary algorithms and compared to the controlled results as appropriate. Either one or both methods have shown variability in their ability relating to the underlying mechanism of carbapenem resistance. Several reports of carbapenemase detection using phenotypic based methods in *Acinetobacter* species, *Pseudomonas* species and Enterobacteriaceae have been documented [90,91]. Reference MIC levels are more sensitive in determining resistant breakpoints for carbapenem susceptibility by broth microdilution and agar dilution, according to Patel et al. [92], than E-test, disc diffusion and many automated systems. For instance, a number of automated systems studied by Queenan and Bush [37] identified up to 87% of KPC-producing *Klebsiella pneumoniae* study isolates as being susceptible to imipenem or meropenem.

The determination of ertapenem MICs by automated testing tends to identify cases which are ertapenem-resistant, which in effect is recommended as a sensitive laboratory test of KPC production irrespective of the method employed [93]. Carbapenem susceptibility breakpoints against meropenem and imipenem keep on changing in recent times, due to lack of global consensus. For instance, previous MIC breakpoints European Committee on Antimicrobial Susceptibility Testing (EUCAST) established were ≤2 µg/mL and >8 µg/mL as susceptible and resistant respectively [19], compared with the Clinical and Laboratory Standards Institute (CLSI) breakpoints of susceptible at ≤4 µg/mL and resistant at ≥16 µg/mL which may compromise results when testing on non-fermenting *Acinetobacter* species [94]. Perez and van Duin [68] established that detection with the new MIC lower breakpoints without using a phenotypic test such as the carbapenem-EDTA combination tests or modified Hodge test may result in lack of differentiation between various mechanisms of carbapenem resistance. For reliable results, EUCAST [95] and CLSI [96] revised MIC breakpoints (Table 1).

The use of chromogenic agar preparation has emerged for identification of MDR organisms from surveillance cultures such as CHROMagar KPC and CHROMagar™ *Acinetobacter* (CHROMagar, Paris, France). Culture media are made selective by adding chromogenic substrates and agents that inhibit growth of other Gram-positive, Gram-negative and yeast isolates. The CHROMagar™ culture media were originally formulated for the screening of patients in the ICU and later redesigned so that *Acinetobacter* species appear as bright salmon red colonies. A new formulation, with the addition of ‘*Klebsiella pneumoniae* carbapenemase supplement’ was able to pick out carbapenem-resistant *Acinetobacter baumannii*. Recent modifications to CHROMagar *Acinetobacter* have improved selective growth for organisms that are resistant to carbapenems [97,98]. According to Arnold et al. [53], CHROMagar KPC has a sensitivity of 100% and specificity of 98.4% compared with polymerase chain reaction (PCR). This technique is used for confirmatory identification of KPC production though is expensive in terms of cost and is mostly performed in research laboratories [53].

The modified Hodge test has been used extensively and is a phenotypic technique for detecting carbapenemase activity routinely used in clinical pathology laboratories. This test was recommended by the CLSI in 2009 [99], however, it is not specific for the detection of all carbapenemase enzymes, most significantly with isolates showing weak positive results and AmpC producers. Bonnin et al. [100] performed modified Hodge tests on 19 carbapenemase-producing *Acinetobacter baumannii* isolates and found negative results for all tested NDM-producing isolates and only weak positive results for VIM-, IMP- and OXA-type producers [100]. There are reports related to susceptibility testing in which the tests showed reduced susceptibility but give consistently negative results for the presence of carbapenemases. These may be due to greater genetic exchange among NDM-1 bacterial strains especially and using non-zinc-supplemented Mueller-Hinton agar medium [101,102].

Double-disc synergy testing has several versions; carbapenem with -clavulanate, -cloxacillin -EDTA or 2-mercaptoproionic acid, and one which utilises a double sided E-test (BioMérieux, Solna, Sweden), imipenem versus imipenem with EDTA, and are used as a screening test for MBL producers. This method is efficient for detection of MBL carbapenemases with high resistance, but may be deficient for detecting MBL producers with low resistance to imipenem according to Walsh et al. [103]. Other results have shown sensitivity with the imipenem-EDTA disc method, 95.7% for *Acinetobacter* species and 100% for *Pseudomonas* species [37,104]. A recent study by Nordmann et al. [32] indicated there is no inhibition test available for detection of OXA-48/OXA-181 producers and observed that EDTA by itself has inhibitory action against some bacteria, due to permeability of the outer membrane, and can lead to false-positive results [32]. According to a recent report from Public Health England [50], resistance to any of the carbapenem drugs may be confirmed using inhibitor based tests. For the detection of carbapenemases in a community, it may largely depend on the availability of existing β-lactamase inhibitors [50].

The newly developed Carba Nordmann-Poirel test, derived from the name “Carbapenemase Nordmann-Poirel”, is in use to detect carbapenemase producers in Enterobacteriaceae. The technique requires no expertise, is reproducible, inexpensive and may easily be adapted as an additional important test in any clinical laboratory. The test may help healthcare facilities to curb and implement rapid containment measures to limit the spread of carbapenemase producers in hospitals [32]. Nordmann et al. [105] described the test as most efficient as compared with molecular-based methods. The Carba NP test is performed in wells and colour change from red to orange or yellow indicates the tested strains are producing carbapenemases. The Carba NP test rapidly and reliably identifies carbapenemase producers by changes in pH values using phenol red as the indicator. Colour develops within 2 h detecting strains that are imipenem resistant due to non-carbapenemase-mediated mechanisms, such as combined mechanisms of resistance or from strains that are carbapenem susceptible but express a broad-spectrum β-lactamase without carbapenemase activity. Reports have shown that the test is 100% sensitive and specific for Enterobacteriaceae [105] and 100% specific and 94.4% sensitive for *Pseudomonas* species harbouring carbapenemases [106]. In contrast, a study by Tijet et al. [107] observed low performance of Carba NP test and false-negative results in OXA-48-like *Pseudomonas aeruginosa* and Enterobacteriaceae isolates with only 80% specificity [107]. Another study reports that Carba NP test, however, cannot differentiate among carbapenemase classes, particularly, weak carbapenemase activity in strains harbouring GES-type carbapenemases in high prevalence areas, Brazil and South Africa. An updated version designated “CarbAcineto NP test” for identifies all the types of carbapenemase in *Acinetobacter* species with high-level of sensitivity and specificity [108,109]. A recently developed carbapenemase biochemical test, the Rapidec Carba NP has been shown to have an identical sensitivity (96%) with the The Carba NP test but a lower specificity (93%) [110]. Compared to The Carba NP test (CNP) and the modified Hodge test, the Rapidec Carba NP is quicker and easier to perform [111].

### 5.2. Problems in Phenotype Based Detection

The recent changes in the breakpoints for carbapenems [112] have further added to the complexity of dealing with multidrug-resistance in the Enterobacteriaceae family. Many laboratories in the USA conform to CLSI standards, approval must come first from this body before clinical laboratories can use the recommendations to detect carbapenemase producing isolates. On the other hand, most European, Asian and African countries use EUCAST or CLSI as their standards for their detection of carbapenemase producing isolates. Delays in breakpoint recommendations on automated kits and devices for use resulted in some laboratories not following strictly laid down rules, hence, undetected CRE with lower breakpoints silently disseminated KPC-producing *Klebsiella pneumoniae* in a long-term facility for children and young adults [113].

During the period of transition, there may be inconsistencies between laboratories regarding identification of ESBLs and CREs. Many different laboratory interpretations of MIC levels may have led to confusion among clinicians about when it is suitable to use an extended spectrum cephalosporin drug, a carbapenem, or neither of them. These determinants are also important for infection prevention and control practices. Pharmacists need to be responsive to these issues so that they can offer the best advice and other method in recommending appropriate antimicrobial choice. Pereira et al. [114] offered suggestions on amended interdepartmental communication within hospital-care services during such transition periods to improve laboratory detection of ESBLs and CREs [114].

### 5.3. Genotype Based Techniques

Molecular techniques have become an efficient tool for carbapenemase detection. Polymerase chain reaction performed on colonies may give results within 4 to 6 h with excellent sensitivity and specificity and this may reduce the chance of spreading organisms in hospitals. Wang et al. [115] reported a real-time PCR assay with specificity and sensitivity at 100% as compared with 90% phenotypic KPC activity when assessed by modified Hodge test (MHT) and sequencing. More recently, multiplex PCR and microarray techniques for detection of several carbapenemase genes in one test have been produced. These are mostly focused on the detection of genes in Enterobacteriaceae including their subgroups of carbapenemases. The limit of these modern tools lacks sequence similarity to genes already described [19,37,116]. Earlier studies largely focused their energies on KPC detection. With the emergence of equally important genes also spreading widely on plasmids, NDM, VIM and IMP in Class B group and recent underestimated Class D serine carbapenemase OXA-48 which is weakly detected by phenotypic methods have revolutionised for different molecular techniques. These are to improve the detection of unknown, dominant resistance genes and their variants to control sporadic dissemination into health-care facilities and community settings [117]. Notwithstanding the enormous contributions of molecular-based assays, they have their own drawbacks and frustrations to end-users mainly due to lack of trained personnel to properly man the equipment, labour-intensive, time-consuming and cost of all consumables and reagents. As reported in two previous studies by Naas et al. [118] and Nordmann et al. [32], molecular assays that are employed should be made as inexpensive, specific, sensitive as possible for the detection of the desired gene and rapid to detect β-lactamase resistance genes to avoid ambiguities of results to improve care-receivers outcome [32,118].

In relation to clinical laboratory practice, innovations of molecular techniques have been evaluated and are currently in use for the detection of carbapenemase producers. Check-MDR CT_102_ DNA microarray, Check direct CPE assay and Hyplex-Super-Bug ID kit are on the market for diagnostic testing purposes. Excellent detection ability and reproducibility with these assays have been reported [118,119,120]. A recent molecular based method demonstrates an intriguing finding in which a self-generating plasmid harbours the OXA-48 gene in multiple transposons Tn1999 rapidly and silently disseminating as one clone [121].

A study by Sparbier et al. [122] described the use of matrix-assisted laser desorption ionisation time-of-flight mass spectrometry (MALDI-TOF MS) as a new tool detecting resistance patterns in bacteria from fresh positive blood cultures. The laser-based ionisation technique can detect changes in mass to charge ratios. Carbapenam resistant bacteria use β-lactamases to physically disrupt the structure of β-lactam antimicrobials. The changes in the mass of the drugs make the resistant bacteria detectable by the MALDI-TOF MS technique, and return results within 4 to 5 h. In another study by Sakarikou et al. [110], MALDI-TOF MS method was used to rapidly detect carbapenemase-producing *Klebsiella* isolates from blood cultures (BCs) and to obtain proteomic profiles enable to discriminate between carbapenemase-producing and non-carbapenemase-producing strains. MALDI-TOF MS is available in most research laboratories for confirmation of a host of bacterial resistance enzymes, with sensitivity and specificity of 98.9 and 97.1% respectively [123]. However, cost and requirement of trained experts to man the instrument limits its use in routine laboratories for diagnostic purposes [121]. Cost analysis of MALDI-TOF MS showed that use of the equipment equated to a net savings of $69,108.61, or 87.8%, in reagent costs annually compared to traditional methods. When total costs are calculated to include technologist time and maintenance costs, traditional identification would have cost $142,532.69, versus $68,886.51 with the MALDI-TOF MS method, resulting in a laboratory savings of $73,646.18, or 51.7%, annually by adopting the new technology [124].

Several molecular techniques have been developed for characterisation of bacterial pathogens including CRE during period of outbreaks and for use in routine clinical laboratories. Notable among these techniques are multilocus sequence typing, multilocus enzyme electrophoresis and DNA fingerprinting methods such as pulsed field gel electrophoresis, amplified fragment length polymorphism and multiple locus variable-number tandem repeat analysis [125]. Whole Genome Sequencing (WGS) has already been used for the characterisation of bacterial isolates in several large outbreaks in several countries and, in the near future, is likely to replace currently used bacterial typing schemes as it provides better resolution of bacterial isolates [126]. Nevertheless, WGS is considered too laborious and time-consuming. Additionally, it is uncertain how genome sequences should be examined for epidemiological characterisation. In future, the key lessons obtained from currently used bacterial typing schemes will enable scientists to condense the WGS data into epidemiologically useful information [127].

## 6. Epidemiology of Carbapenemase-Producing Organisms

Across the globe, first reports of carbapenemases occurred in the 1980s. Its subsequent spread raised a number of concerns, and these have coincided with years in which no new antimicrobial agents against Gram-negatives have been developed. Japan reported the first carbapenemase from an *Aeromonas hydrophila* isolate in the 1980s. Sequentially, followed in London (1982) by Seoul imipenemase (SME-1) from *Serratia marcescens*, imipenemases (IMI-1) in California (1984) and NMC-A in France (1990), both from *Enterobacter cloacae* [40].

Carbapenem resistance involves one or more of several diverse mechanisms of actions in multi-resistant Gram-negative bacteria. The most frequently encountered β-lactamase enzymes are the acquired carbapenemases in the Ambler class A group of which *Klebsiella pneumoniae* carbapenemases predominate worldwide [116,128,129,130,131]. An alarm call on the spread of carbapenem-resistance has been in existence since KPC was identified in 1996 in the United States. Clinical characteristics of KPC carrying organisms vary with local differences and conditions. Countries such as Israel, Greece and Colombia have outbreaks of KPC through their existing local situations and others mainly by importation for instance, in Canada, Australia and New Zealand [73,132]. Following worldwide spread of *Klebsiella pneumoniae* ST258 type strain, and throughout the USA in particular, MLST, a specific molecular tool was used to find out the global epidemiology linkages and help understanding the dissemination of KPC genes. There are fresh fears and challenges on-going and under discussion by researchers as to how to curb the spread of carbapenem resistance across the globe for epidemiological reasons. Detection has been difficult due to differences in their genetic makeup within and across countries. Researchers have observed divergent resistant patterns from CREs in Europe, the United States and South America. The unusual resistance factors have been monitored through a surveillance programme, named the Intensive Care Antimicrobial Resistance Epidemiology (ICARE) by the CDC from 2000 onwards. Earlier CRE infections, particularly KPC, were confined to hospitals in New York. Between 2000 and 2010, the infection rate had increased from 1% to 12% in 42 States respectively [133] with a 50% associated mortality rate [32,134]. Thirty-nine states of the USA, including Puerto Rico, were hit by the threat of KPC positive organisms within the next few years. Strict control measures were instituted, such as short-stay in acute-care settings, made the CRE infections infrequent and contained the rate below 5% as compared with 8% in long-term acute-care hospitals monitored by the CDC Network [135]. The occurrence resulted in many other KPC-positive *Klebsiella pneumoniae* isolates to be categorised while the majority have been found in *Pseudomonas aeruginosa* [136], *Pseudomonas putida* and others in the Enterobacteriaceae family [137].

Studies have shown that the spread of KPC-positive organisms in some parts of the USA was not related to travel. Most infections seemed to occur in hospitals, with the majority of the outbreaks contracted in long-term care facilities (LTCFs) for care receivers with severe medical conditions who needed longer stays for their clinical management [135,138,139]. Patel et al. [140] reported 38% infection-specific mortality and 48% in hospital mortality for patients infected with carbapenem-resistant *Klebsiella pneumoniae* isolates, contrasting with 12% and 20% for carbapenem-susceptible *Klebsiella pneumoniae* respectively [140]. The clinical impact of KPC-positive bacteria occurs within 48 h of admission, with isolates identified from samples such as blood, wound swabs, urine and sputum. Patients with a history of frequent exposure in a health-care facility may easily trigger community-acquired infections [52,135]. The guidance as to the detection and control of these isolates have been expanded with appropriate recommendations as a public health measure in 2012 by CDC on regional bases all over USA [135,141].

Currently, 39 countries in the European Union including France and the United Kingdom have come together to fight the burden of CREs in their respective countries. The study is being coordinated by the European Centre for Disease Prevention and Control in Stockholm to keep epidemiological data of patients who test positive for such bacteria in their health-care institutions [142,143]. Between the years 2003 and 2007, the UK experienced the first recorded KPC gene with a KPC-4 variant in an *Enterobacter* species from a blood sample and the first case of a known *Klebsiella pneumoniae* producing KPC-positive isolate, all from Scotland [56,144]. There were scattered increases in numbers as the KPC-positive isolates continued to be identified by the UK national public reference laboratory in 2008 (4 hospitals 5 isolated) and in 2009 (12 hospitals 13 isolated) with *Klebsiella pneumoniae* ST258 the most frequent strain to be captured by MLST (10 out of 12). The source was traced to Greece, Cyprus or Israel, with patients having travelled to those countries in the time prior to admission. Between 2010 and the first six months of 2012, the epidemiological pattern rapidly changed in terms of numbers identified, 231 KPC-positive strains in 2010, 368 in 2011 and 293 for the first six months in 2012 were referred for further confirmation at the national reference laboratory [8,128].

Rapid spread of CREs across Europe was observed in late 2005 when an identified KPC isolate in Israel was later found to be genetically related to a type strain in New York. France acquired its first KPC-2-positive *Klebsiella pneumoniae* in 2005 from a patient who had had 3 months admission in a New York City (NY, USA) hospital. The strain type was isolated from blood and urine cultures. From 2009 to 2012, there have been several CREs investigated. In a recent study, five cases out of 20 were confirmed and linked their sources to countries such as Kuwait, China, Italy and Israel. Community acquired strains were found to be rare and most of the documented cases in France were from persons colonisation with the strains [73,145].

Carbapenemases became a public health concern in 2007 when a total of 1275 CRE infections were identified across their health care centres. KPC-positive organisms found its way in to other countries including Italy, Colombia and the United Kingdom [11,146,147]. In early 2008, Sweden was the next country to identify multidrug-resistant KPC-positive isolates, this time with a different metallo-β-lactamase enzyme. More cases of this type of enzyme were seen within three years in the USA and in the UK. Other Enterobacteriaceae, such as *Escherichia coli* were harbouring NDM, exhibiting more resistant traits than KPC-positive *Klebsiella* isolates [148]. There were no clear-cut patterns of dissemination for carbapenemase positive *Klebsiella* bacteria including those with NDM-1 genes into hospitals. For instance, following a Colorado episode, the CDC used whole-genome sequencing, a technology utilised for the first time to resolve outbreaks in the hospital. Researchers were in doubt as to exactly how NDM was escalating among the population. Studies later revealed that bacteria carrying the NDM enzyme found its way outside the boundary of hospital settings into community water and sewage environments with some epidemiological link to parts of Southern Asia [11,149].

In Italy, the enzyme type described in Israel ST258 isolates located in transposon Tn4401 was detected in KPC-positive isolate (KPC-3 gene type) [150]. Over the years, 7 different patients with wound infection in Verona hospital at the surgical ICU had their isolates closely related to KPC-3-positive *Klebsiella pneumoniae* ST258 [151]. The situation was more disturbing when in 2011, the same strain type carrying colistin-resistance was found in Palermo general hospital in different acute wards and elsewhere from isolates (*Acinetobacter baumannii* and *Pseudomonas aeruginosa*) in other countries [152,153]. Spain also confirmed a KPC-3 variant among 8 suspected cases through colonisation in a hospital in Madrid from a Spanish man in his mid-sixties in 2009, and 7 of the isolates were ST384 while the other one was carrying ST388 type [154]. A later outbreak which occurred in late 2009, this time a variant closely related to the KPC-2 gene type had been found in three *Citrobacter freundii*. The earlier isolates, none were attributed to travels outside the country [155]. However, in close neighbour Portugal, a KPC-2-positive *Escherichia coli* isolate was recovered from river water although there were no reports of medical cases in connection of KPC-positive isolates in recent times from their hospitals [156].

Poland has a history of high prevalence of CREs carrying KPC genes. A KPC-positive case was confirmed in Warsaw. Later, a number of cases were confirmed by the national reference laboratory in five Warsaw hospitals towards the end of 2008, with more identified from 2009 to 2012 (not published). Most common sources of infections were either through stool or urine and ST258 type of strains were high among the isolates countrywide [157,158]. In Greece KPC-positive *Klebsiella pneumoniae* isolates were confirmed in Sweden and France in 2007 [159,160]. An outbreak of KPC-2-positive *Klebsiella pneumoniae* was reported afterwards in a tertiary hospital in Heraklion [161], and two years later the type strain had spread into all tertiary hospitals including most acute-care services countrywide [162,163].

Currently, KPC-positive organisms are frequently encountered in Greece and are not only found in the ICU, patients on surgical and medical wards have been victims of such infections. Outbreaks in long-term care facilities have been described, *Escherichia coli* carrying KPC-2-positive gene contributed to 40% infections outside ICUs [164]. However, there has been no evidence of horizontal transmission into the community. On the other hand, several confirmed cases of colonisation of KPC-positive *Klebsiella pneumoniae* from Greece have been reported in other countries most of them following international travel [165]. There have been several other carbapenemase enzymes established in most KPC-positive isolates in Greece. A surveillance study involving 40 hospitals had 5% VIM-1 or VIM-4 genes and the whole study team all reported 378 KPC-positive isolates in their findings. Other studies have revealed much higher numbers in VIM isolates or the presence of both VIM and KPC in most isolates. Of all the epidemic cases, Greece had recorded most KPC-positive isolates having other genes linkages, and ST258 type included with the genetic make-up closely related [166,167].

Israel is one such country with a successful story of controlling CRE infections after strain ST258 was suspected to have been imported from the USA in 2005 and eventually spread over all Israeli health-care facilities [168]. Most contributing risk factors at that period included poor infection control set-up, previous use of antimicrobial and ICU stay. Until middle of 2008, outbreak of KPC-positive Enterobacteriaceae had been controlled considerably, with incidence reduced by 79% in the country and bacteraemia taking up 50% as the attributable mortality rate of all clinical infections [31,169]. Acquiring CRE infections from the community was not encountered, however, in long-term care facilities (LTCFs) the carriage was well established in a study carried out by Cantón et al. [147] due to frequent transfer of patients from acute-care facility to the latter for further management. One of the measures taken to control the spread was compulsory search of CREs from rectal samples in high risk patients [62,147].

The most hit areas of the Asian continent by the CRE isolates were India, Hong Kong, Taiwan, South Korea, and central, north-west, eastern and south-west parts of China. In India in particular, KPC-positive isolates were observed during a phase-3 clinical trial of tigecycline drug between 2002 and 2006 from three organisms; *Klebsiella pneumoniae*, *Proteus mirabilis* and *Escherichia coli* [170]. By the end of 2010, an active surveillance study identified 9 more organisms harbouring the *bla*KPC gene in which 8 *Klebsiella pneumoniae* isolates were with 7 KPC-2 and 1 KPC-3, and 1 *Escherichia coli* isolate with KPC-2. Interestingly, one of *Klebsiella pneumoniae* isolates was co-harbouring KPC-2 with OXA-1, NDM-1, TEM-1, CTX-M-15 and SHV-12 β-lactamases, and RmtB known to be transferring aminoglycoside resistance [171]. The SENTRY programme carried out (between 2006 and 2007), identified OXA-181 as the gene recognisable in Indian health-care facilities [101]. Earlier KPC-positive organisms seemed to be rare until French researchers reported KPC-positive *Escherichia coli* suspected to have been imported from India by a patient who had been colonised [172].

The most common carbapenemase gene in China is KPC-2 type found in a number of *Klebsiella* species. Zhejiang province recorded KPC-positive *Klebsiella pneumoniae* in China in the year 2004 from a mid-seventy-year-old ICU patient [173], and then significant numbers of KPC-positive Enterobacteriaceae were later identified in the eastern part of China. As many as 95 isolates of *Klebsiella pneumoniae* that showed susceptibility to carbapenem agents were later found to be carrying KPC-2 genes, closely related to ST258 type [174]. The Shanghai region detected KPC-3 genes, one each in *Citrobacter freundii* and *Escherichia coli* respectively and infections were mainly from urine and sputum specimens. In many instances, the KPC-3 gene is more common in *Enterobacter cloacae* and *Klebsiella pneumoniae* isolates found abroad [175]. China had no effective infection control strategy in place and most patients received broad-spectrum antimicrobials including carbapenems in ICU wards where many patients had indwelling medical procedures complicating management. The most alarming situation was the detection of KPC-positive *Enterobacter cloacae* and *Citrobacter freundii* in hospital sewage, raising awareness of possible pollution of water bodies and reservoirs [176] while Ge et al. [177] studied and reported eminent community acquired infections with KPC-2-postive isolates in China.

Endemicity of KPC genes had been identified in these regions of Latin America, specifically, across Colombia among the Enterobacteriaceae family. Reported cases of KPC-positive isolates of KPC-2 type were found in *Klebsiella pneumoniae* isolates from two patients in 2005 [178,179], and both had no travels outside the country. Later in 2006, the country became the first to experience a KPC gene in *Pseudomonas aeruginosa* [180]. After two years, a KPC-3-producing *Klebsiella pneumoniae* outbreak occurred in which 20 died out of 32 patients. Fourteen of the death tolls were due to infections, with one patient travelling from Israel, a country with several outbreaks of KPC-3 genes [181]. A number of KPC genetic lineages were identified in Colombia; namely *Pseudomonas aeruginosa* ST235, ST308, ST1006 and ST1060, while in *Klebsiella pneumoniae*, ST258 and ST51233 were identified [73,182]. Subsequent reports on molecular investigations revealed extensive dissemination of KPC genes spreading across 3 regions out of 7 metropolitan areas studied in 2009 [179,183]. Argentina also had its KPC-2-positive *Klebsiella pneumoniae* identified in 2006.

By the end of 2010 following SENTRY studies carried out for 2 years, substantial increases in KPC-positive isolates in Argentina and Brazil were recorded [184]. In another surveillance study conducted in 7 cities, 65 out of 514 *Pseudomonas aeruginosa* isolates were carrying KPC genes, and most had genetic relatedness to ST654, different from the ST258 *Klebsiella pneumoniae* type which suddenly spread up to mid-2009 in Argentina [185,186]. A KPC-positive enzyme in Brazil was recorded in 2006, a variant KPC-2-positive from 4 *Klebsiella pneumoniae* isolates in an intensive care unit with no trace of overseas travels [187]. Subsequent expanded studies carried out across the country including waste water from hospital environment, revealed clone type-ST437 was common among KPC-2-harbouring *Klebsiella pneumoniae* isolates [188]. Brazil and other parts of the region had most species of *Pseudomonas* harbouring KPC-2 genes from hospital sittings. Venezuela and Chile, in particular, had no evidence of endemicity, however, several outbreaks of KPC-positive carriers from the Enterobacteriaceae group were latterly recorded [183].

Many countries have not instituted active surveillance studies specifically monitoring the KPC threat since its inception around the globe, the majority of which are in the African continent. Countries in Africa, including Morocco, Kenya and South Africa have reported NDM-1 as the most dominant carbapenemase gene [189,190]. However, South Africa was the first to have reported a KPC-2-positive organism in 2012 [191]. In Ghana, a recent study by Codjoe [192] showed that KPC was absent in all 111 carbapenem resistant Gram-negative bacteria that were screened; the carbapenemase genes identified were *bla*NDM-1 in *Acinetobacter baumannii*, *bla*VIM-1 in *Pseudomonas* species and *bla*OXA-48 in *Klebsiella pneumoniae*. In Ghana, multi-drug resistance of Gram-negative bacteria has reached alarming rates [193,194,195,196]. Strict infection control measures and regular surveillance programmes instituted by countries such as Australia and New Zealand have recorded prevalence as low as 1% of KPC harbouring genes in hospital acquired Enterobacteriaceae infections. The control measures were implemented after increased numbers of care-servicers were accepted to be treated from the Bali bombing episode in 2002 and the 2004 tsunami disaster, which occurred in the Indian Ocean [73,135]. Recently, China has launched a national action plan for combating various antimicrobial resistance threats including CREs, which promises to make a substantial impact on public health, both locally and globally.

## 7. Treatment Options for CRE Infections

Antimicrobial treatment of CRE infections has been challenged by the emergence of more complex resistance phenotypes as well as economic and regulatory pressures. Agents such as polymyxins and tigecycline have recently seen resurgence in their clinical usage (particularly colistin) in the management of multidrug-resistant Gram-negative infections, particularly CRE including carbapenem-resistant *Acinetobacter baumannii* in most hospitals [197].

The polymyxins are active agents and attain sufficient serum levels in the treatment of serious bloodstream and CRE infections. The agents produce additive or synergistic effects on humans against multidrug-resistant organisms including *Acinetobacter baumannii* isolates when combined with another antimicrobial agents such as tigecycline. In a study conducted by Lee et al. [198], 16 patients with recurrent infections caused by KPC-producing *Klebsiella pneumoniae* evaluated that three out of twelve managed with polymyxin monotherapy experienced polymyxin resistance in their treatment. None of the four cases which were treated with polymyxin in combined therapy with tigecycline detected resistance to either antimicrobial agent [198,199]. Out of several outcomes of monotherapies, polymyxin B monotherapy exhibited inferiority as against combination therapy in the management of patients [200]. In an outbreak of KPC-2 infections in Greece, 22% resulted in polymyxin failure after treatment [161].

Tigecycline, a glycylcycline, is active in vitro against most carbapenem-resistant *Escherichia coli*. The drug is licensed for most complicated intra-abdominal, skin and soft tissue infections. Interestingly, a study reports success in various infections caused by carbapenemase producers [53]. A later review of 10 studies including 33 patients with serious infections caused by multidrug resistant-Enterobacteriaceae, Kelesidis et al. [201] reported 70% favourable outcomes with tigecycline treatment of cases, while 49% were only for intra-abdominal infections. Recurrence of infections was detected, prompting the management of the drug for a longer period in order to attain favourable results, due to delayed clearance of the CRE strain from the body which several studies had reported [201]. However, tigecycline use alone remains a concern in blood, urine, respiratory or other serious infections, and there have been several reports of development of resistance due to low concentration level [202,203].

Combination therapy with tigecycline proved successful in many instances with positive clinical outcomes in the majority of infections involving KPC bacteria [201]. During an outbreak of KPC-2 infections in Greece, 88% of the patients were effectively treated with combination therapy involving tigecycline [53,161]. In a study involving over 1000 patients with severe illnesses and a high prevalence of multidrug-resistant organisms, tigecycline showed a significant success and a positive tolerability profile when the drug was used [60,204].

Aminoglycosides, notably gentamicin, amikacin and tobramycin have different in vitro activities. Treatments of CRE infections may depend on the susceptible organism as studies have shown gentamicin activity against gentamicin-susceptible strains in urinary tract infections have positive outcomes. Of the aminoglycosides, amikacin appears to be the more active against CREs when compared with gentamicin or tobramycin [19]. While in another study, amikacin and tobramycin showed remarkably low susceptibility to infections caused by MDR Gram-negative bacteria. This may be due to gentamicin modifying enzymes which have been carried by these MDR organisms. The use of aminoglycosides as monotherapy against carbapenemase-producing *Klebsiella pneumoniae* infections are considered ineffective, and therefore not recommended for clinical management of patients [60,205].

Fosfomycin, a bactericidal antibiotic that inhibits bacterial cell wall biogenesis has seen its use renewed globally in response to the recent threat of antimicrobial resistance including carbapenem-resistant *Klebsiella pneumoniae* isolates [206,207]. The drug is effectively used to treat urinary tract infections and has low rates of resistance. However, poor outcomes may occur when treating complicated *Pseudomonas aeruginosa* as a urinary pathogen. Many patients that developed treatment failure were immunosuppressed or had urethral stents due to the use of fosfomycin as monotherapy in kidney transplant cases.

Combination therapies have shown remarkable outcomes in dealing with MDR and CRE infections. The most commonly used combinations are colistin, polymyxin B or tigecycline combined with a carbapenem. In a retrospective study by Qureshi et al. [208] on patients with bacteremia, the monotherapy, either tigecycline or colistin-polymyxin B alone had 58% mortality rate as compared with 13% for the tigecycline or colistin-polymyxin B combined with a carbapenem on a 28-day assessment, and this was observed in infections caused by KPC-producing *Klebsiella pneumoniae* isolates [208]. Lee and Burgess [209] also studied several antimicrobials for treatment of patients and recommended combination therapy (polymyxin plus tigecycline, polymyxin plus carbapenem, polymyxin plus aminoglycoside) as the best option compared with monotherapy (tigecycline or colistin-polymyxin B) in complicated infections involving MDR and CRE [209]. In future, treatments for infections caused by carbapenemase producers may involve new β-lactamase inhibitors such as methylidene penems; avibactam, MK-7655; the maleic acid derivative ME1071; (‘neoglycoside’) plazomicin, a novel aminoglycoside; the polymyxin derivatives NAB739 and NAB7061; and the siderophore monosulfactam, BAL30072 combined with cephalosporins and novel antimicrobial agents that are effective against these pathogens such as solithromycin and omadacycline [10,203,210]. Continual studies as regards to combination therapy trials need to be intensified until more efficient and acceptable combined therapy is accepted and recommended for the management of CRE infections.

## 8. Future Solutions to Curb Global CRE Threat

The threat must be answered by strict adherence to infection control strategies and policy guidelines designed for individual countries [75,105]. Performing more efficient identification of carbapenemase producers in the clinical microbiology laboratory is an important first step and provides key evidence for the control of CRE infections. The early identification of carbapenemase-producing isolates both in clinical infections and at the carrier-state should be mandatory to prevent the development of untreatable infections [18,41]. The right to obtain a prescription at the first sign of a trivial infection propels the threats of resistance globally. Therefore, international campaigns to educate health-care providers, patients and lay persons may be warranted to limit the over-use and abuse of antibiotics in humans and agriculture. A recent systematic review indicated that, CPE outbreaks can be controlled using combinations of existing measures [211]. Optimizing the timeliness of control measures and better understanding of which control measures are most effective will enable resources to be allocated wisely and reduce transmission.

Development of novel antimicrobials remains the prime objective for CRE control, a look at critical interventions aimed at preventing the transmission and infections with these organisms are of great significance [18,41]. In waiting for more effective treatment options, healthcare settings can use prudent antimicrobial stewardship and effective infection control methods to decrease the impact of these organisms where this emergence of carbapenem resistance is known to be uncommon. Heightening the awareness of the public health threat by governments, policy makers, and or taking political decisions based on active surveillance testing for CRE may have some impact and support health caregivers to curb the resistance.

## 9. Conclusions

The growing threat to public health includes the rapid spread of CRE into the community. Multidrug-resistant Gram-negative bacteria such as *Acinetobacter* and *Pseudomonas* species, less encountered in hospitals are the main organisms spreading into the community. These organisms have the potential to spread resistance to other bacterial isolates into the community. Despite efforts to control carbapenem resistance, a definite solution to the problem is still far from achievement. Carbapenemase-encoding genes are already widespread in certain parts of the world particularly, Europe, Asia and South America while the situation in other places such as sub-Saharan Africa is not well documented. There is the need for active surveillance of carbapenemase-encoding genes as major step to controlling the menance.

## Figures and Tables

**Table 1:** Breakpoint values of minimum inhibitory concentration (mg/L) for carbapenems according to guidelines in Europe (EUCAST) and the United States (CLSI).

	EUCAST						CLSI					
	*Enterobacteriaceae*		*Acinetobacter*		*Pseudomonas*		*Enterobcteriacae*		*Acinetobacter*		*Pseudomonas*	
Carbapenem	S	R	S	R	S	R	S	R	S	R	S	R
Doripenem	≤1	≥4	≤1	≥2	≤1	≥2	≤1	≥4	≤2	≥8	≤1	≥8
Ertapenem	≤0.5	≥1	-	-	-	-	≤0.5	≥2	-	-	-	-
Imipenem	≤2	≥8	≤2	≥8	≤4	≥8	≤1	≥4	≤2	≥8	≤2	≥8
Meropenem	≤2	≥8	≤2	≥8	≤2	≥8	≤1	≥4	≤2	≥8	≤2	≥8

EUCAST: European Committee on Antimicrobial Susceptibility Testing (www.eucast.org/clinical_breakpoints), version 7.1, 2017; CLSI: Clinical and Laboratory Standards Institute, 27th ed. CLSI document M100S 27, 2017; S: sensitive; R: resistant.
